# Sexual healthcare and at-home STI test collection: attitudes and preferences of transgender women in the Southeastern United States

**DOI:** 10.3389/fpubh.2023.1187206

**Published:** 2023-05-31

**Authors:** Olivia T. Van Gerwen, Erika L. Austin, Carly W. Bethune, Patrick S. Sullivan, Christina A. Muzny

**Affiliations:** ^1^Division of Infectious Diseases, University of Alabama at Birmingham, Birmingham, AL, United States; ^2^Department of Biostatistics, UAB School of Public Health, Birmingham, AL, United States; ^3^School of Medicine, University of Alabama at Birmingham, Birmingham, AL, United States; ^4^Rollins School of Public Health, Emory University, Atlanta, GA, United States

**Keywords:** at-home testing, qualitative research, sexually transmitted infections, transgender, sexual health

## Abstract

**Background:**

HIV and sexually transmitted infections (STIs) disproportionally affect transgender women in the United States, particularly in the Southeast where rates of HIV and bacterial STIs are especially high. Despite the high HIV/STI burden among transgender women, their engagement in sexual healthcare services, including HIV/STI testing, is low. Understanding reasons for this disconnect is essential in developing HIV/STI prevention efforts for this population, especially in the Southeastern US, where access to affirming sexual healthcare providers and resources is limited. We aimed to perform an exploratory qualitative study to describe the attitudes and preferences of transgender women living in Alabama with regards to sexual healthcare and at-home STI test collection.

**Methods:**

Transgender women ≥18 years old residing in Alabama were invited to participate in virtual individual in-depth interviews via Zoom. The interview guide explored participant experiences engaging with sexual healthcare services as well as preferences related to extragenital (i.e., rectal, pharyngeal) and at-home STI testing for gonorrhea and chlamydia. A trained qualitative researcher coded transcripts after each interview and iteratively amended the interview guide as themes emerged. Data were coded and thematically analyzed using NVivo qualitative software.

**Results:**

Between June 2021-April 2022, 22 transgender women were screened and 14 eligible women enrolled. Eight participants were white (57%), and six were black (43%). Five participants (36%) were living with HIV and engaged with HIV care services. Interview themes included preference for sexual healthcare environments specializing in LGBTQ+ care, enthusiasm toward at-home STI testing, an emphasis on affirming patient-provider interactions in sexual healthcare settings, a preference for sexual healthcare providers involved in STI testing who were not cisgender men, and gender dysphoria around sexual health discussions and testing.

**Conclusion:**

Transgender women in the Southeastern US prioritize affirming provider-patient interactions, however resources in the region are limited. Participants were enthusiastic about at-home STI testing options, which have the potential to mitigate gender dysphoria. Further investigation into development of remote sexual healthcare services for transgender women should be performed.

## Introduction

In the United States, it is estimated that between 14% and42% of transgender women are living with HIV (1, 2). Despite this immense disease burden, uptake of effective HIV prevention strategies such as Pre-Exposure Prophylaxis (PrEP) remain low in this population, especially in the Southeastern US (3). Reasons for this are multifactoral, including limited access to gender-affirming healthcare providers and services, intersecting stigma associated with PrEP and transgender identity, and concern for drug-drug interactions between PrEP and hormone replacement therapy (4, 5). Furthermore, the Southeastern US accounts for the most of the new HIV infections annually and is home to a growing population of transgender individuals (6). Thus, a better understanding of how to increase PrEP uptake among transgender women in the Southeastern US is essential in efforts toward ending the HIV epidemic.

One crucial component of PrEP care is routine bacterial STI (i.e., gonorrhea, chlamydia, syphilis) screening, because individuals taking PrEP experience an increased incidence of STI infection and bacterial STIs are also common among transgender women (7–9). The risk of HIV transmission may double in the setting of a bacterial STI co-infection, making frequent STI screening a priority for patients on PrEP to optimize its efficacy (10). Depending on sexual practices, extragenital STI testing (i.e., testing at rectal and/or pharyngeal sites) for bacterial STIs is also an important consideration for transgender women taking PrEP. Despite this, clinicians frequently limit specimen collection to urogenital sites, which can miss a large proportion of extra-genital STI infections (11).

Routine STI testing is also an essential part of sexual healthcare for transgender women living with HIV. A retrospective cohort study found that chlamydial infection from extra-genital sites in 312 transgender women living with HIV accounted for 80% of positive results and extra-genital gonorrhea accounted for 82% of positive results (12). Given that the sexual behaviors of some transgender women (i.e., receptive anal intercourse) may lead them to be at risk of extragenital STIs, developing inclusive and acceptable means of STI screening/testing in this population are essential to promote engagement in HIV prevention and sexual healthcare services.

Access to trans-affirming STI screening/testing is not only an essential component of PrEP care for transgender women, but is also a necessary service for transgender women who are sexually active, regardless of their HIV status. Unfortunately, a major barrier to access to sexual healthcare for this population is the stigma and discrimination transgender women often experience in healthcare settings (13). In the Southeastern US, these issues are further compounded by limited sexual healthcare resources serving LGBTQ+ populations. As such, transgender women may have unique preferences related to STI screening/testing in this setting. In a pilot study previously conducted by our team (4), transgender women in the Southeastern US identified STIs to be a major concern in their communities. In the current qualitative study, we explored community-specific considerations among transgender women living in Alabama regarding receipt of sexual healthcare services including STI screening/testing in general and as a part of HIV PrEP care.

## Methods

The purpose of this exploratory qualitative study was to describe the experiences of transgender women living in Alabama. This study asked how transgender women's general experiences in health care settings shape their openness to seeking sexual health care as well as their perceptions of at-home STI testing, given their previous health care experiences. A phenomenological approach was adopted for this study due to the limited information available in the existing literature that describes transgender women's experiences with sexual health care in their own words (14).

Eligible participants were ≥18 years old, identified as transgender women (i.e., assigned male at birth and identify as female) and resided in the state of Alabama. Exclusion criteria included not speaking English and being unable to participate in an individual virtual interview using Zoom (because the study was conducted during the COVID-19 pandemic). Given both the high prevalence of HIV within this population as well as the high STI burden experienced by transgender women, regardless of HIV status, all transgender women were allowed to enroll. Participants were recruited through flyers posted in locations around the Birmingham and Huntsville, Alabama metropolitan areas, including local clinics and community organizations serving transgender individuals, bars, clubs, art galleries, and locations across the University of Alabama at Birmingham (UAB) campus. Participants were also recruited via referrals form clinicians at the UAB Gender Health Clinic (GHC). Participants received a $50 Visa gift card for their participation in the study.

This study was approved by the UAB Institutional Review Board (IRB) (protocol #IRB-300005085). Participants provided verbal consent, including consent for audio recordings, before beginning of the in-depth individual interviews. The participants were emailed a copy of the consent form prior to the interview for review. Participants verbally consented with study staff who documented this on a printed version of the consent form. This consent process was approved by the UAB IRB and was completed prior to participation in any study-related activities.

### In-depth interviews

After verbal informed consent and permission for audio recording were obtained via Zoom, participants completed an interviewer-administered socio-demographic survey, which included questions on age, race/ethnicity, insurance status, sexual history, STI history, details regarding gender transition, and history of PrEP use. Transgender women living with HIV were not asked questions pertaining to PrEP.

**Figure 1:**
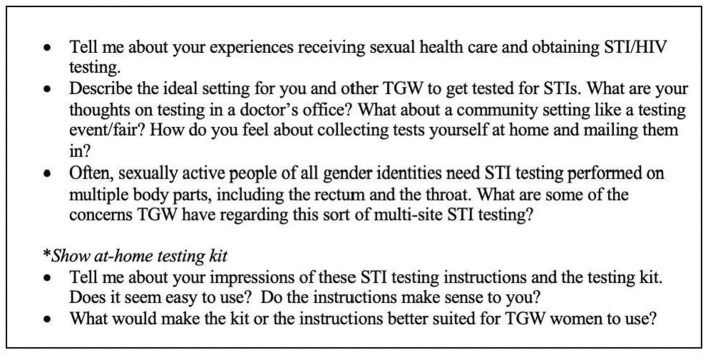
IDI script.

Following completion of the questionnaire, an in-depth individual interview (IDI) was conducted using a script to facilitate discussion (Figure 1); the interviewer (author OVG) is a cisgender women and sexual health care provider with 9 years of experience working with LGBTQ+ populations. Participants were asked about their experiences in sexual health care settings, particularly related to obtaining HIV/STI testing. They were also be asked about their impressions of at-home self-collected STI testing kits and materials, specifically the acceptability and appropriateness for use by transgender women. Sample at-home self-collected STI testing kits, including the collection materials and instruction documents, were also shown to participants (Supplementary Figure 1). These kits included extragenital and urine specimen collection materials as well as lancets and dried blood spot cards to be used for HIV testing. This was both to provide a visual reference for participants for this type of testing and to ensure that all instructions and infographics were acceptable for use by transgender women. All interviews were audio recorded via Zoom and uploaded to UAB Box on an encrypted, password-protected computer. They were later transcribed verbatim by a professional transcription service.

### Qualitative data analysis

A descriptive phenomenological approach was used in this work (15, 16). The initial in-depth interview script was developed based on the lead author's experience as a physician providing sexual health care to transgender women; this script was iteratively revised throughout the data collection process to explore emerging themes and to further examine conflicting perspectives. Memos of emerging issues and discussions within the research team were used to document the initial stages of data analysis. Once data collection was completed, the lead author conducted a review of all interview transcripts and began developing thematic codes. Using NVivo qualitative software, all transcripts were coded; these initial codes were further reviewed by a second member of the research team (author ELA) to ensure reliability and to check for potential bias resulting from the lead author's identity as a health care provider (17).

## Results

### Sample characteristics

Between June 2021 and April 2022, 22 transgender women were screened for eligibility in this project. Those who were not enrolled were either lost to follow up or no longer wished to participate when they were contacted to schedule an IDI. A total of 14 transgender women enrolled in the study and completed an IDI. Sociodemographic characteristics of the enrolled participants are described in Table 1. The mean age of participants was 33 years old (SD 10 years). Eight participants were white (57%), and six were black (43%). Five participants (36%) were living with HIV and all of them were engaged with HIV care services. Participants reported a variety of genders of their sexual partners, with the most reporting sex with cisgender men (71.4% [*n* = 10]). Of note, participants could select multiple options when reporting the genders of their sexual partners. In addition to genders of sexual partners, participants reported the following sexual orientations: gay/homosexual (*n* = 7), pansexual (*n* = 5), bisexual (*n* = 1), straight/heterosexual (*n* = 1). More than half reported having a history of an STI (*n* = 8) and 28.6% (*n* = 4) reported having participated in transactional sex at some point in their lives. One participant reported having a gender-affirmation surgery, which was an orchiectomy. Of the participants who were not living with HIV (*n* = 9), all had heard of PrEP, but only one (11.1%) reported a history of PrEP use.

**Table 1:** Sociodemographic data for transgender women participants in the Southeastern U.S. (*n* = 14).

	***N* (%) or Mean ±SD**
Age	33 ± 10 years
**Race/ethnicity**	
Black, Hispanic	1 (7.1%)
Black, non-Hispanic	5 (35.7%)
White, non-Hispanic	8 (57.1%)
**Gender of sexual partners**	
Transgender women	4 (28.6%)
Transgender men	2 (14.3%)
Cisgender women	5 (35.7%)
Cisgender men	10 (71.4%)
Genderfluid individuals	1 (7.1%)
**Self-reported sexual orientation**	
Gay/homosexual	7 (50.0%)
Pansexual	5 (35.7%)
Bisexual	1 (7.1%)
Straight/heterosexual	1 (7.1%)
Self-reported STI history	8 (57.1%)
Living with HIV	5 (35.7%)
Participation in transactional sex	4 (28.6%)
Currently using HRT	11 (78.6%)
History of gender-affirmation surgery	1 (7.1%)

HIV, human immunodeficiency disease; HRT, hormone replacement therapy; SD, standard deviation; STI, sexually transmitted infection.

### Major themes

#### Preference for sexual healthcare environments specializing in LGBTQ± care

Participants reported that care environments with specialization in LGBTQ+ healthcare services were preferred when pursuing their sexual healthcare needs. Given this type of provider expertise in queer health, participants reported more affirming experiences than with other types of healthcare providers. They cited trauma associated with going to clinical spaces in general, particularly related to being blatantly discriminated against by staff at all levels (i.e., receptionists, nurses, physicians, etc.). Many recounted experiences they had had in non-LGBTQ+ specializing healthcare venues as being cold and insensitive.

“*[the workers at non-LGBTQ*+ *focused clinics] are just not educated on trans people or gay people, but I just feel like they're insensitive and just like rude. Like even when they took my blood they kind of just like stuck me and it was very like aggressive; it wasn't like empathetic or anything like that.”*

“*So [at a local LGBTQ*+ *focused clinic], I'm not as worried about because I don't feel threatened, but if I were outside [that clinic in the main healthcare system] like in a different hospital room, you know, and it was something real sensitive like I was doing bottom surgery or discussing something about that may be different on a cis person I would rather not be in a room with someone overhearing all that.”*

“*I would say by virtue of being at [a local LGBTQ*+ *focused clinic], like I just feel like it's sort of like a self-selecting thing like you're not going to be volunteering or working there if you have negative ideas about trans people, you know, which is what sort of led me to wanting to go there and, you know, maybe even deal with a less efficient and profession, I guess, like operation because I knew like no matter what nobody's gonna look at me weird, everybody's gonna be like affirming and accepting…”*

Many participants reflected specifically on negative experiences in non-LGBTQ+ focused care environments related to being misgendered, often intentionally, by clinic staff.

“*So I kept getting called ‘he', I kept getting called by my real name. It was like they was stating facts about me that I already knew. It was just… I don't know, it was just… it wasn't a pleasant… pleasant experience.”*

“*Interviewer: So they misgendered you*.

*Respondant: A lot, even after it was corrected they still… well, they was like, well, I'm goin' by what I see in the computer and what I see in the books. But I'm like… but look at me… you know, but-…*

*Interviewer: Yeah, I'm a person in front of you, telling you that's not my name.”*

They also endorsed appreciating the LGBTQ+ representation in these focused care environments. Particularly, they acknowledged feeling more comfortable receiving sexual healthcare in a place with visible signs of transgender allyship (i.e., flags, posters, displays of staff pronouns) as well as openly LGBTQ+ staff. LGBTQ+ representation was seem as reassuring that participants would be cared for in an affirming manner.

“*…it's as simple as like just seeing the pronouns in someone's bio or like their display name or something, just in and of itself like, you know, virtue signaling in like a good way.”*

“*But they have been super welcoming. When you first walk in the area… the lobby is very welcoming. There's two queers behind the counter doing all the intake and paperwork, so I automatically feel very, uh… one of them is actually trans feminine, so that, you know, made me feel a lot more comfortable.”*

#### Enthusiasm toward at-home STI testing

When presented with the option of self-collection of STI test specimens in the home, namely for urogenital and extragenital gonorrhea and chlamydia, participants were enthusiastic. They also cited that the kits seemed easy to use. Several participants did acknowledge, however, apprehension around performing STI testing that requires a finger stick using a lancet or needle (i.e., syphilis and HIV testing). However, they cited both privacy and convenience as appealing aspects of an at-home testing option. Participants expressed that their desires for these attributes came from a long history of mistrust of the medical system and fear of being in physical danger in sexual healthcare settings. In addition, participants expressed that the option of at-home test collection removes the need for transgender women to discuss the details of their anatomy, sexual behaviors, or other potentially uncomfortable details directly with a healthcare provider.

“*I hate to say it, but like especially with the trans community, we have like typically such a large distrust with doctors because… or the medical field in general, that if we are given the slightest chance to do it ourselves instead and like be maybe within the comfort of our own homes or whatever to do that kind of thing, we will do that because that is much nicer than like having to go out and do it.”*

“*I personally would like doing it either at home or doing it myself and… just don't wanna be like naked around a stranger and how much of it is, you know, would actually be a worry of some sort of like negative interaction because I was trans or something.”*

“*…people are nervous and anxious when it comes to sharing the intimacy of those details with people they don't know, especially a medical provider, and, you know, just the physical of having someone swab in places like that is a little discomforting. I personally think that at-home testing could be and may as well, already is, revolutionary in terms of making sure people are being tested correctly.”*

Participants also stated that having access to at-home STI testing provided a sense of empowerment around sexual health promotion. They stated that they may be more likely to utilize this resource than if an at-home testing option were not available.

“*And it would be convenient to know what type of testing you can have done, whether you need to go through a doctor or whether it's something you could approach the clinic and just have simply done yourself, and then have that information readily disseminated out into the population in some sort of public awareness.”*

#### Emphasis on affirming interactions between providers and patients in sexual healthcare settings

Participants not only sought to receive sexual healthcare services in LGBTQ+-focused care environments, but also emphasized the importance of certain characteristics of the clinician-patient relationship. Preference was expressed for providers who demonstrated warmth and passion about their job. It was important to participants to feel genuinely cared for by their providers. This was especially important given the potential for discussion of sensitive topics such as sexual history and behaviors as well as exams involving genitalia. Some participants discussed negative experiences they had had in sexual health care settings which involved staff being rude and judgmental, especially when a visit was specifically for STI testing. Participants preferred providers who treated sexual health care and STIs as normal parts of the human experience as opposed to stigmatizing patients who may present for such testing.

“*… I feel like that they actually do care about your health instead of just doing their job; it's more than just their job to them. They actually do care.”*

“*And it does mean a lot to me to be able to go into a doctor's office and be able to have a conversation with the person who's sticking needles in me and taking my blood instead of just kind of it being a very clinical thing and then I leave.”*

“*I feel like in a field like [sexual health] you kind of have to be empathetic and compassionate and not like rude, and a lot of them are just very rude. Like going [to a local health department] I just feel like you walk in and just all eyes on you, like, oh, they have an STD, and it's a very uncomfortable environment, at least for me.”*

“*So I feel like at UAB they make you feel human and like, you know, things happen. It's not like, oh, you did this to yourself; it's just, you know, stuff happens, gotta move forward, we'll teach you, you know, what to do to keep yourself protected, give you condoms.”*

#### Preference for sexual healthcare providers who were not cisgender men

When having STI testing done in the clinic setting, participants overwhelmingly preferred female providers to male providers. In fact, they cited that one of the benefits of being able to do at-home, self-collected STI testing was avoiding encountering male health care providers or clinic staff. Several themes emerged as to why female providers were preferred. One was that transgender women feel sexually fetishized by men, even in clinical settings. Participants expressed unease with the potential for a male provider deriving sexual pleasure from providing sexual health services such as STI testing to transgender women patients.

“*We are typically seen as like only doing it for sex or sexual pleasure or whatnot, and so it kind of, um… we kind… we, again using we instead of me… I kind of fear that like a cisgender heterosexual man might feel that way about me and think that he can use me for sexual pleasure.”*

“*…not because I necessarily would think that they're a bad person, but in the back of my head, trans women in particular are fetishized in pornography and it's a large percentage of online pornography consumption, and even in a clinical environment in the back of my head I would probably have this question of is this some kind of weird thrill for them.”*

Participants also expressed a preference for female providers for several reasons. They noted that, in general, female providers tend to have a more natural understanding of the female experience. Several participants also cited that female providers tend to be more affirming and understanding in terms of gender diverse patients.

“*Respondent: No, with a female doctor it's not a problem, but with a male, yeah*.

*Interviewer: Okay. Tell me more about that. What are the concerns there?*

*Respondent: My concern is a male doctor … they don't understand what I go through. Now with a woman, they're more like… like they understand, they do. And they talk to me more better because they can relate to me what I'm stressin' about, but with a male I become scared because they don't know what I go through, they don't know how I'm feelin', they don't know half the stuff I've dealt with.”*

“*Respondent: … I would always feel more comfortable with like a woman doing it, yeah*.

*Interviewer: Okay. Can you speak more as to why that is?*

*Respondent: Yeah, maybe just my own personal biases, honestly, I just generally that expect of the two options that the odds are the woman is going to be more understanding and it's more likely that she's either going to be like directly affirming or just also has experiences as a woman and so it's more likely that it will be a positive interaction from my experience.”*

#### Gender dysphoria exacerbated by sexual healthcare encounters

Gender dysphoria associated with various components of receiving sexual healthcare were also described by participants. Feelings of dysphoria around sexual healthcare environments were viewed as negative and, therefore, made participants apprehensive about engaging with such services. Specifically, participants cited conversations referring to tests in a gendered way that were particularly dysphoric. For example, labeling testing materials for men or for women based on the test needing to be performed on a penis or a vagina, respectively, was not preferred. Instead, emphasis was placed on making testing and sexual health discussions center around a patient's specific anatomy. Participants also said that discussing their genitalia and anatomy on an as-needed basis only was important to them. They said that if such information was not needed to provide optimal sexual health care, then discussions of their genitalia should be avoided. Reasons for this included the acknowledgment that many transgender people experience genital dysphoria if they have not yet undergone any gender-affirming surgeries and that clinicians inquiring about their genitalia are often viewed as curiosity on the behalf of the provider as opposed to necessary for their medical care.

“*…One of my worries… and it goes back to the anatomy and stuff… some part of that interaction sort of like triggering some sort of dysphoria, like I guess I would say the big thing is not saying … this is the test for women or something like that… [but instead] maybe making it anatomy specific and not gendered at all.”*

“*I think trying to strip away all of that until it becomes absolutely necessary, so, you know, what do you need to be tested for… let them describe the symptoms instead of asking them do you have this coming out of this, because that person sitting in front of you might not have those, and now you've just put them in an uncomfortable position because what if they have genital dysphoria and you've just really hammered home that you thought they had so and so.”*

Participants also acknowledged that sexual health care differs from other sorts of routine care in its content and that because of this, transgender patients may feel more discomfort or dysphoria discussing their bodies or having invasive examinations performed that involve their genitalia.

“*I think a lot of trans people to start with, even before transition, are uncomfortable with their bodies and so they may be more hesitant to take part in exams that are more personal and more invasive than the standard tongue depressor in your mouth sort of a thing, only because there's that extra level of dysphoria that you may not be dealing with, with a regular patient.”*

## Discussion

This study augments previous work done by our team exploring the preferences of transgender women in Alabama regarding how they receive sexual healthcare (4). In the wake of the COVID-19 pandemic, telehealth and at-home healthcare technologies are now a permanent fixture in care delivery, both in the US and worldwide (18). Acceptability and development of such technologies for PrEP and STI testing have been promising in recent years (19–22). Thus, understanding how these options can be implemented to suit the unique needs and preferences of populations who may access PrEP or frequently need STI testing, including transgender women, is essential.

In this study, a sample of transgender women in Alabama expressed insights regarding best practices in the provision of sexual healthcare services to their community. Specifically, they highlighted the need for genuine relationships with affirming providers in LGBTQ+ dedicated spaces as well as enthusiasm (excitement) for at-home STI testing. Their reasons for desiring at-home STI testing underscore several ways in which health care in the Southeastern US is currently failing this population. The transgender women in our study desired to test at home because of privacy and the ability to avoid potential negative interactions with health care staff and providers. Previous studies of providers working with transgender patients, including those with HIV, have noted perceived barriers including lack of care accessibility and security, providers' misunderstanding of the transgender community, and lack of cultural competency of information systems and staff (23). Stigma toward this community, particularly transgender women living with HIV, is also prevalent (24). Although participants acknowledged the existence of some exemplar gender-affirming care providers in the region, such resources are limited in the state of Alabama. The results of our study should serve as a call to action for improved provision of gender-affirming care for transgender people, including sexual health services. Most notably, a patient-centered approach to transgender care where patients are treated with compassion and understanding is desired by patients, especially in sexual healthcare settings. In addition, given the need for routine STI testing for patients on PrEP, these approaches may also serve to increase uptake of PrEP and its consistent use among this population.

Participants also cited that the sample at-home STI testing kits appeared to be both easy to use and acceptable for use among transgender women. Overall, when developing such resources, investigators and manufacturers should avoid using gendered language in the patient facing instructional materials used for testing. Community involvement and vetting of language and schematics should be prioritized. Stakeholders of the transgender experience offer the most accurate and informed perspective on how such materials should be developed.

Finally, these results highlight the need for comprehensive, gender-affirming healthcare services for transgender people in the sexual healthcare setting. This not only entails the clinician-patient interactions that take place, but the overall culture and atmosphere of clinical spaces in which transgender women receive care. Representation and celebration of transgender identity by clinic staff at all levels play an integral role in patients engaging in sexual healthcare services. While at-home testing for STIs is a promising and important option for patients, there are still times when patients need to access in-person services. Enhanced medical education and LGBTQ+ cultural competency training for all providers and staff are key first steps to creating such a culture. Enhancing patient engagement in sexual health services through the creation of such environments will help to combat the STI epidemic faced by gender diverse populations.

This study had several limitations. Given that interviews were conducted during the COVID-19 pandemic, our recruitment period was delayed and IDIs were conducted virtually over Zoom. While use of Zoom was helpful in terms of including transgender women from various parts of the state outside of the Birmingham, AL metropolitan area and allowing participants to discuss sensitive topics in the comfort of their homes where they felt the safest, the dynamics of in-person interactions were missing. Specifically, detailed reading of body language cues and more interactive demonstration of testing kits was not possible. One important point that was not discussed in these IDIs was the role of cost regarding at-home STI testing. There were some mentions from participants that affordable testing was preferred, but this theme was not explored in this study and should be investigated in future work. We also recognize that the healthcare landscape for transgender individuals in Alabama is not nationally or globally representative. Currently, the transgender people and the healthcare providers caring for them in Southeastern US experience unique culture and legal challenges in providing gender-affirming healthcare that are clearly reflected by the limited resources and in-person interactions expressed by participants. These include ongoing legislative efforts aimed at limiting, banning, or criminalizing the provision of gender-affirming healthcare for transgender individuals in this area (25, 26).

In conclusion, transgender women prioritize affirming provider-patient interactions; however, gender-affirming sexual health resources in the Southeastern US are limited. Given these regional limitations and enthusiasm for at-home STI testing options, further investigation into developing remote sexual healthcare services for transgender women are of interest.

## Data availability statement

The raw data supporting the conclusions of this article will be made available by the authors, without undue reservation.

## Ethics statement

The studies involving human participants were reviewed and approved by University of Alabama at Birmingham Institutional Review Board. Written informed consent was obtained from all participants for their participation in this study and was approved by the University of Alabama at Birmingham Institutional Review Board.

## Author contributions

Study conception and design: OV, EA, PS, and CM. Data collection and draft manuscript preparation: OV and CB. Analysis and interpretation of results: OV and EA. All authors reviewed the results and approved the final version of the manuscript.
