# Supplementary material for: Sexual healthcare and at-home STI test collection: attitudes and preferences of transgender women in the Southeastern United States
Source: Front Public Health. 2023 May 31;11:1187206. doi: 10.3389/fpubh.2023.1187206 (PMC10264611; doi:10.3389/fpubh.2023.1187206)
Supplement: Supplementary file 1 [file Data_Sheet_1.PDF]

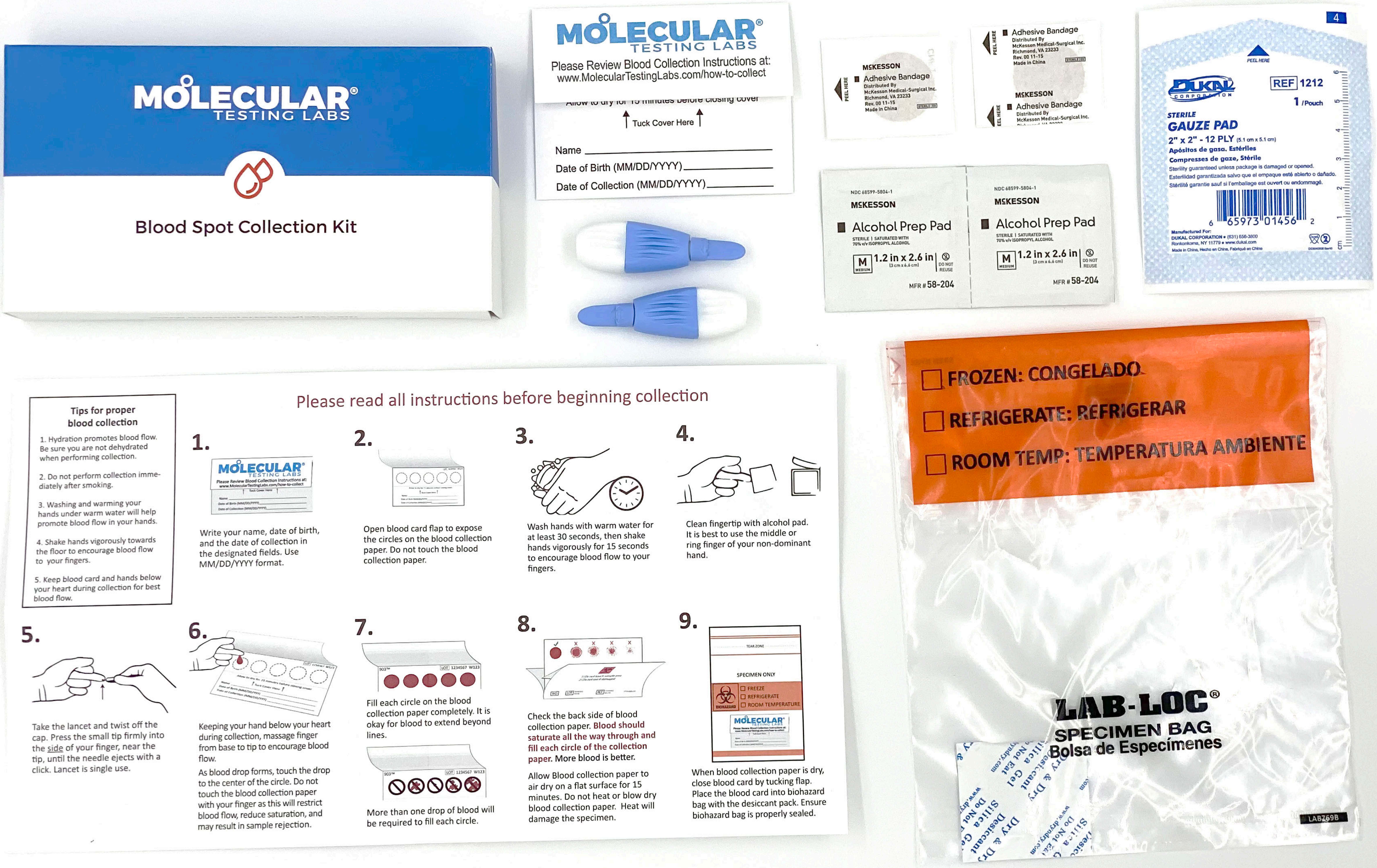

MOLECULAR® TESTING LABS
Blood Spot Collection Kit
MOLECULAR® TESTING LABS
Please Review Blood Collection Instructions at:
www.MolecularTestingLabs.com/how-to-collect
Tuck Cover Here
Name
Date of Birth (MM/DD/YYYY)
Date of Collection (MM/DD/YYYY)
MCKESSON
Adhesive Bandage
PEEL HERE
MCKESSON
Alcohol Prep Pad
1.2 in x 2.6 in
MFR # 58-204
MCKESSON
Alcohol Prep Pad
1.2 in x 2.6 in
MFR # 58-204
PEEL HERE
DUKAL CORPORATION
REF 1212
1 /Pouch
STERILE
GAUZE PAD
2" x 2" - 12 PLY
Apósitos de gasa. Estériles
Compresses de gaze, Stérile
Sterility guaranteed unless package is damaged or opened.
Esterilidad garantizada salvo que el empaque esté abierto o dañado.
Stérilité garantie sauf si l'emballage est ouvert ou endommagé.
6 65973 01456 2
Please read all instructions before beginning collection
Tips for proper blood collection
1. Hydration promotes blood flow. Be sure you are not dehydrated when performing collection.
2. Do not perform collection immediately after smoking.
3. Washing and warming your hands under warm water will help promote blood flow in your hands.
4. Shake hands vigorously towards the floor to encourage blood flow to your fingers.
5. Keep blood card and hands below your heart during collection for best blood flow.
1. Write your name, date of birth, and the date of collection in the designated fields. Use MM/DD/YYYY format.
2. Open blood card flap to expose the circles on the blood collection paper. Do not touch the blood collection paper.
3. Wash hands with warm water for at least 30 seconds, then shake hands vigorously for 15 seconds to encourage blood flow to your fingers.
4. Clean fingertip with alcohol pad. It is best to use the middle or ring finger of your non-dominant hand.
5. Take the lancet and twist off the cap. Press the small tip firmly into the side of your finger, near the tip, until the needle ejects with a click. Lancet is single use.
6. Keeping your hand below your heart during collection, massage finger from base to tip to encourage blood flow.
As blood drop forms, touch the drop to the center of the circle. Do not touch the blood collection paper with your finger as this will restrict blood flow, reduce saturation, and may result in sample rejection.
7. Fill each circle on the blood collection paper completely. It is okay for blood to extend beyond lines.
More than one drop of blood will be required to fill each circle.
8. Check the back side of blood collection paper. Blood should saturate all the way through and fill each circle of the collection paper. More blood is better.
Allow Blood collection paper to air dry on a flat surface for 15 minutes. Do not heat or blow dry blood collection paper. Heat will damage the specimen.
9. When blood collection paper is dry, close blood card by tucking flap. Place the blood card into biohazard bag with the desiccant pack. Ensure biohazard bag is properly sealed.
FROZEN: CONGELADO
REFRIGERATE: REFRIGERAR
ROOM TEMP: TEMPERATURA AMBIENTE
LAB-LOC®
SPECIMEN BAG
Bolsa de Especímenes
LAB269B
Dry & Desiccant
Silica Gel
Do Not Eat

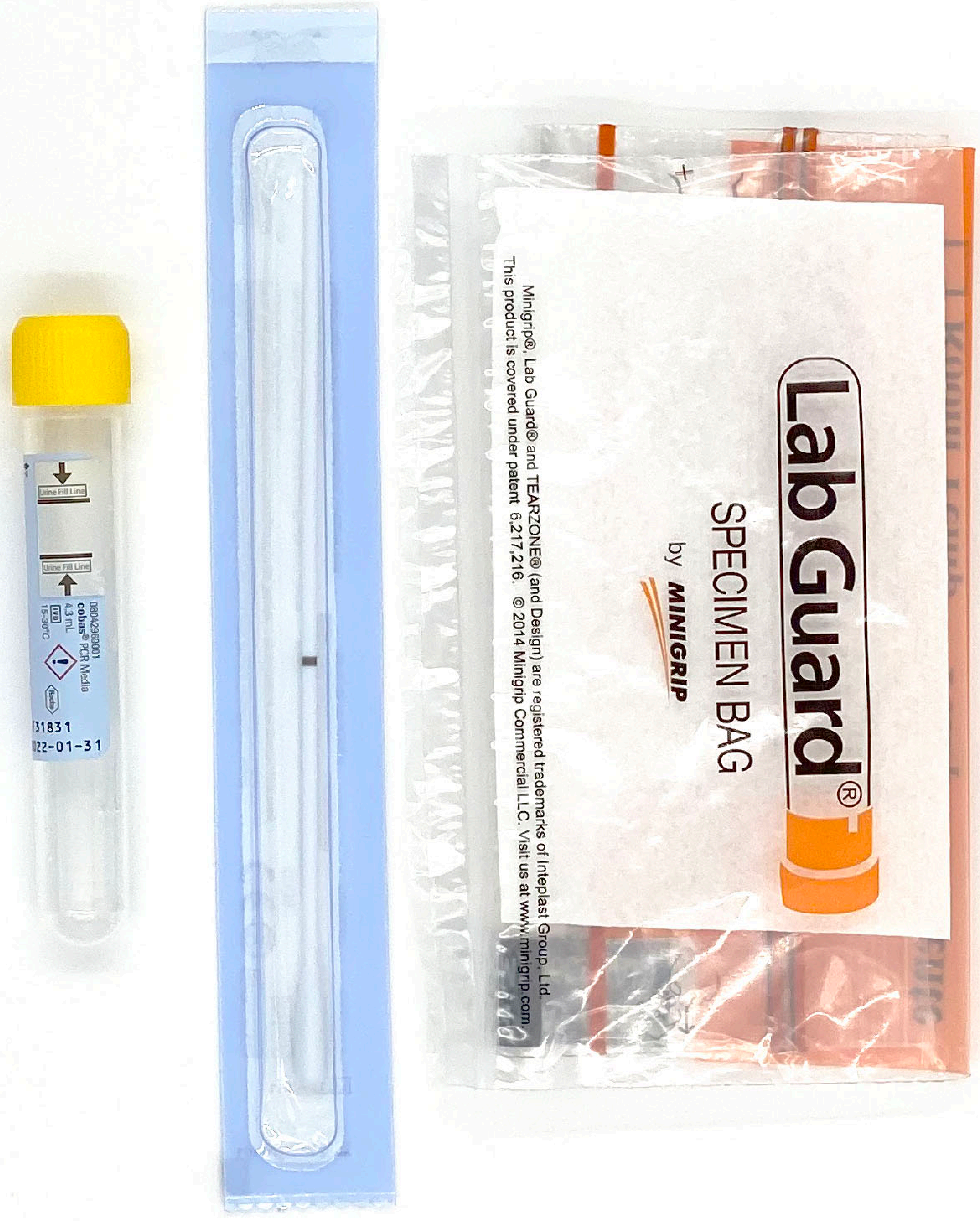
Urine Fill Line
Urine Fill Line
cobas® PCR Media
4.3 mL
15–30°C
Lab Guard®
SPECIMEN BAG
by MINIGRIP
Minigrip®, Lab Guard® and TEARZONE® (and Design) are registered trademarks of Inteplast Group, Ltd.
This product is covered under patent 6,217,216. © 2014 Minigrip Commercial LLC. Visit us at www.minigrip.com.

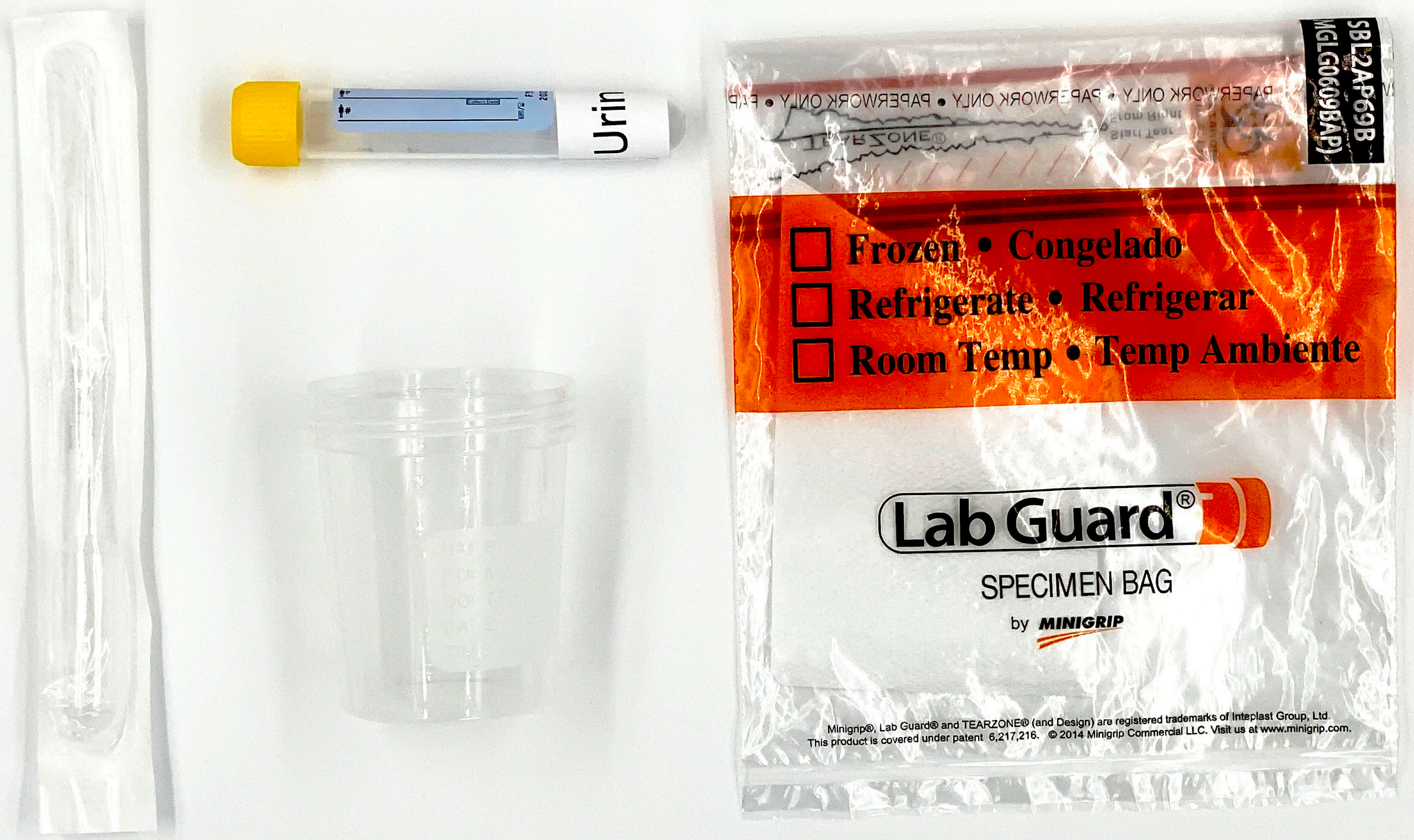
Urin
SBL2AP69B
MGLG0609BAP)
Frozen • Congelado
Refrigerate • Refrigerar
Room Temp • Temp Ambiente
Lab Guard®
SPECIMEN BAG
by MINIGRIP
Minigrip®, Lab Guard® and TEARZONE® (and Design) are registered trademarks of Inteplast Group, Ltd.
This product is covered under patent 6,217,216. © 2014 Minigrip Commercial LLC. Visit us at www.minigrip.com.

URINE

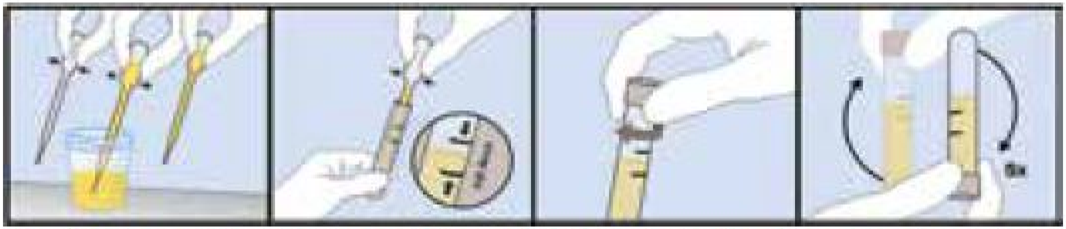

DO NOT FILL ABOVE MAX FILL LINE OR SAMPLE WILL BE REJECTED

1. Do not urinate for at least 1 hour prior to collection. Using first morning urine is best.
2. Urinate directly into the collection cup, and collect 30 mL - 50 mL of urine.
3. Use the pipette to transfer urine from the collection cup into the collection tube(s) marked “Urine”.
4. Fill the collection tube(s) until the combined liquid is between the designated fill lines. **Do not fill tube past the maximum fill line or specimen will be rejected.**
5. Place lid onto the collection tube(s) and ensure it is closed evenly and tightly.
6. Write your date of birth in MM/DD/YYYY format onto the tube in the designated area (DOB).
7. Write the specimen collection date (today’s date) in MM/DD/YYYY format onto the tube in the designated area (Coll. Date).
8. Place specimen into an empty biohazard bag and ensure the seal is closed completely.

ANAL SWAB

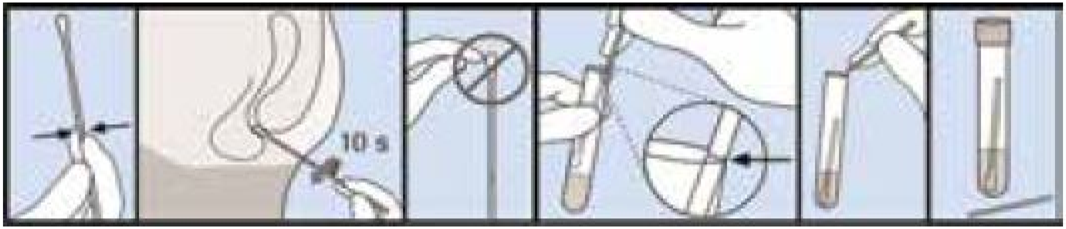

1. Hold the swab with the score line above your hand.
2. Insert the swab 3 - 5 cm (1 - 2 in) into the anal canal.
3. Gently turn the swab for 5-10 seconds while rubbing the swab against the walls of the rectum.
4. Carefully withdraw the swab and place into the collection tube marked “Anal”. Break the swab at the score line by bending against the collection tube.
5. Place lid onto the collection tube and ensure it is closed evenly and tightly.
6. Write your date of birth in MM/DD/YYYY format onto the tube in the designated area (DOB).
7. Write the specimen collection date (today’s date) in MM/DD/YYYY format onto the tube in the designated area (Coll. Date).
8. Place specimen into an empty biohazard bag and ensure the seal is closed completely.

Tips for proper blood collection

1. Hydration promotes blood flow. Be sure you are not dehydrated when performing collection.

2. Do not perform collection immediately after smoking.

3. Washing and warming your hands under warm water will help promote blood flow in your hands.

4. Shake hands vigorously towards the floor to encourage blood flow to your fingers.

5. Keep blood card and hands below your heart during collection for best blood flow.

TO PREVENT REJECTED SPECIMENS, PLEASE READ ALL INSTRUCTIONS BEFORE BEGINNING COLLECTION

1.

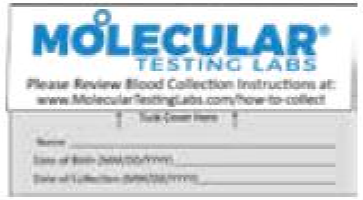

Write your name, date of birth, and the date of collection in the designated fields. Use MM/DD/YYYY format.

2.

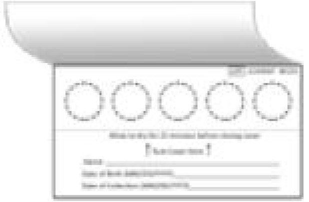

Open blood card flap to expose the circles on the blood collection paper. Do not touch the blood collection paper.

3.

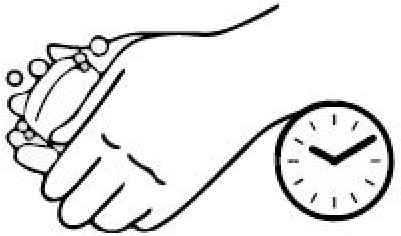

Wash hands with warm water for at least 30 seconds, then shake hands vigorously for 15 seconds to encourage blood flow to your fingers.

4.

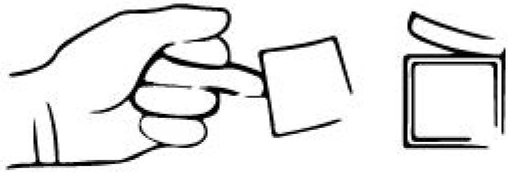

Clean fingertip with alcohol pad. It is best to use the middle or ring finger of your non-dominant hand.

5.

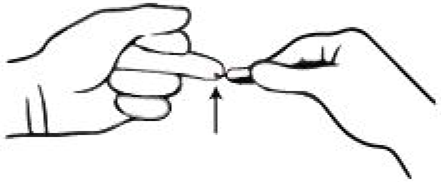

Take the lancet and twist off the cap. Press the small tip firmly into the side of your finger, near the tip, until the needle ejects with a click. Lancet is single use.

Keeping your hand below your heart during collection, massage finger from base to tip to encourage blood flow.

6.

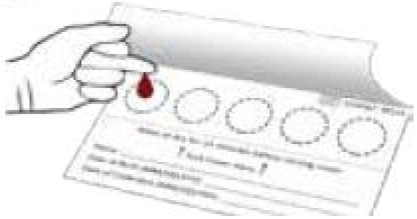

Starting at the center, apply 3-6 drops to fill the circle and soak through the collection paper. Do not touch the paper with your finger as this will restrict blood flow. Once circle is full move on to next circle. It is okay for blood to extend beyond lines. but do not let blood spots spread into each other. **Do not add additional blood to a circle once completed or dry. The "layering" of blood will invalidate the collection.**

7.

YES

Proper Collection.

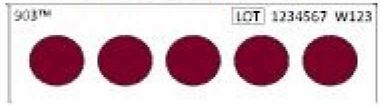

NO

Not enough blood.
Fill circles completely.

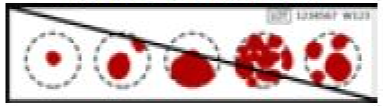

NO

Do not layer blood once the paper has begun to dry.

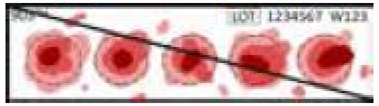

8.

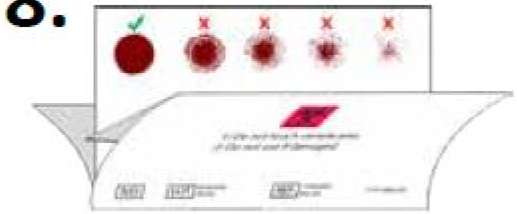

Check the back side of blood collection paper. **Blood should saturate all the way through and fill each circle of the collection paper.**

Without closing the blood card, lay it on a flat surface and allow the blood collection paper to air dry at room temperature, for at least 30 minutes.

Do not heat, blow dry, or expose the blood collection paper to direct sunlight. Heat will damage the specimen.

9.

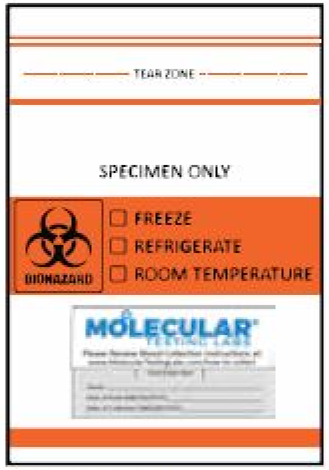

When blood collection paper is dry, close blood card by tucking flap. Place the blood card into biohazard bag with the desiccant pack. Ensure biohazard bag is properly sealed.
